# Book Review: Bye Bye I Love You: The Story of Our First and Last Words

**DOI:** 10.5195/jmla.2025.2146

**Published:** 2025-07-01

**Authors:** Edwin Battistella

**Affiliations:** 1 edbattistella@gmail.com, Southern Oregon University, Ashland, OR

In *Bye Bye I Love You*, Michael Erard presents the results of five years of study about first words, last words, and the parallels between the phenomena. The result is a unique cultural history, erudite and wide ranging, spanning cultures and disciplines. The first six chapters, roughly half of the book, deal with the study of babies' first words arising from studies by linguists and psychologists. Erard tackles questions of epistemology (how do we know what babies are doing when they make word-like expressions?) and he describes the difficulty of the concept “first word,” both linguistically and socially. He also takes us through the history of scholarly attention to first words and their connection to attitudes about childhood and infancy.

Erard discusses contemporary literature on child language, but he has also tracked down many little-known accounts and studies of children communication, including an 1828 book on progressive education by Albertine-Adrienne Necker de Saussure, the great-aunt of the Swiss linguist Ferdinand de Saussure. He shares insights from historian Linda Pollock's study of over four-hundred English-language diaries from the sixteenth to nineteenth centuries. In addition, *Bye Bye I Love You* surveys the cultural practices of various groups, showing how some cultures favor “a prescribed verbal form that human babies must produce as the threshold that grants them personhood” (80) such as reference to *mama* or *papa*, first words with an almost ritual status. By contrast, there are also cultural instances of what Erard calls *laissez parler*, where the baby is just allowed to speak. One classic (literally) example involves from ancient Roman practices, as described by Marcus Terentius Varro: when children developed linguistic agency, however it is manifest, Roman parents would make an offering to Farinus, the little-known god of first words. Finally, Erard takes us through the ways in which interest in infants first words has been connected to speculations about the origins of language itself.

Chapters 7 through 12 are about the end of life. The end of life also has ritualized qualities connected to how cultures think of death and how people think of elders losing their faculties. As was the case with child language, Erard has uncovered some key studies of communication at the end of life. Prominent among these is Sir William Osler's relatively little known “The Study of the Act of Dying,” a never-published account of 486 patients at the Johns Hopkins Hospital between 1900 and 1904, but we also learn about the fifteenth century *Tractatus artis bene moriendi*, a guide for Christian death, and more recent studies such as Karl Guthke's *Last Words* and Maggie Callanan and Patricia Kelley's *Final Gifts*. In addition to consulting texts, Erard has interviewed palliative care physicians and nurses, chaplains, end-of-life doulas, speech-language pathologists, linguists and more. He points out some of the difficulties of studying last words, such as the popular fascination with hagiographical “famous last words.” However, even for those who wish to study last words more systematically, he notes that there are cultural, ethnic, and gender differences in whose last words get documented and how well. In addition, he shows how end-of-life experiences differ widely depending on the causes.

Throughout, Erard focuses our attention on expectations: what is it we expect people to say–to produce linguistically–as they are dying. Central to the discussion is again the contrast between ritual and sincerity. For some cultures and individuals, last words, like first words, are ritualized events, expressions of faith for example. For others, last words are idiosyncratic communications, rooted in individual expressive agency.

Erard has an MA in linguistics and a PhD in English and a suite of academic publications. He is also a journalist, so the scholarship is presented in a readable fashion. The audience for *Bye Bye I Love You* will be quite diverse. Linguistics, sociologists, psychologists, doctors, nurses, and end-of-life doulas will all find value in Erard's sensitive history and first and last words. Of equal value are Erard's interspersed personal reflections on first and last words and on the transition into various states of life. For me, the book certainly sparked contemplation of my language experiences: what were my first words and the baby culture I grew up in (Dr. Spock) and, since I am now of a certain age, prompted thoughts of what is to come (hopefully not immediately). I finished the book excited to be present at the birth of a new academic field—the linguistics of end-of-life communication, and to have witnessed its first words.
